# Meeting abstracts from the 11th European Cytogenetics Conference: late breaking abstracts: Florence, Italy. July 01-04, 2017

**DOI:** 10.1186/s13039-017-0331-7

**Published:** 2017-09-21

## Poster Abstracts

### 1. Clinical Cytogenetics

#### 1.P80 Subtelomere fluorescence in situ hybridization as a powerful tool to screen for submicroscopic balanced rearrangements in couples with recurred abortions/stillbirth despite of the era of exome sequencing

##### Ilona Dietze-Armana^1^, Barbara Fritz^2^, Karl Mehnert^3^

###### ^1^Cytogenetic genetikum Neu, Ulm, Germany; ^2^Clinical Genetics Philipps, University Marburg Germany; ^3^Genetic Counselling/Cytogenetic genetikum Neu, Ulm, Germany

####### **Correspondence:** Ilona Dietze-Armana

Cryptic terminal chromosomal rearrangements are known to cause human genetic diseases. They are mostly undetectable by conventional chromosome analysis even of the highest resolution. Subtelomere fluorescence in situ hybridisation (FISH) analysis has increasingly been used as an adjunct to routine cytogenetic testing in order to detect small rearrangements. Indications for this application are: 1) conspicuous familial medical history, 2) ultrasound abnormalities in a previous pregnancy, 3) late abortion/IUFT/still birth.

Here we present four couples with remarkable medical history. We found in a mother, (case 1), with ultrasound abnormalities in two fetuses, a 10q/11p subtelomeric cryptic reciprocal translocation (crt). In the fifth abortion of a woman (case 2) we found a paternally inherited unbalanced form of the 8q/11q translocation detected by array-CGH. In case 3, a mother, who had a stillbirth at 28 weeks of gestation, a crt 6q/8q was found. Furthermore, a 1q/16p crt was seen in a man whose partner had recurrent abortions (case 4).

The clinical information including the family history of these patients will be summarized and compared with the current literature.

We propose the use of subtelomere FISH as a reliable, rapid and economical first approach to detect unbalanced terminal chromosomal rearrangements, especially in absence of major chromosome rearrangements in routine diagnosis. Detailed analysis of the family history helps to choose further methods of more diagnostic power.

#### 1.P81 The role of classical cytogenetics for detection of autosomal structural changes after birth in the Romanian population

##### Mariela Sanda Militaru^1^, Mihai Militaru^2^, Alexandra Maris^3^, Maria Stefanut^4^, Iulia Blanaru^4^, Diana Militaru^5^, Eleonora Dronca^1^

###### ^1^UMF Iuliu Hatieganu Medical Genetics Cluj, Napoca, Romania; ^2^UMF Iuliu Hatieganu Pediatrics no.2 Cluj, Napoca, Romania; ^3^Children's Hospital Intensive care unit Cluj, Napoca, Romania; ^4^Genetic Center Genetics Cluj, Napoca, Romania; ^5^Faculty of Medicine Student Oradea, Romania

####### **Correspondence:** Mariela Sanda Militaru

**Introduction**

Chromosome abnormalities account for 50% of all spontaneous miscarriages and are present in 0,5-1% of all newborn infants.

The present study was undertaken to investigate the different types of structural chromosomal aberrations and their relative frequencies in a group of patients with suspected genetic disorders in the Romanian population, and to identify precisely the role of cytogenetic investigation in confirming the diagnosis, thus allowing proper genetic counseling.

**Material and Methods**

We cytogenetically investigated a total of 927 patients using the G banding technique for identifying and characterizing the chromosomes of lymphocytes.

**Results**

We identified a total of 121 chromosomal aberrations (13%), of which 83 were numerical abnormalities (8.9%) and 38 were structural abnormalities (4.1%). Of the 38 structural abnormalities, 28 were autosomal chromosomal abnormalities. Changes in chromosome structure encountered were: different translocations-11 cases, deletions-7 cases, inversions-5 cases, 3 cases chromosomal polymorphisms, and 2 cases of marker chromosomes.

**Conclusions**

Detection of structural chromosomal abnormalities is a key element for both correct etiological diagnosis and genetic counseling. The results are comparable to those of similar studies.

#### 1.P82 A case of Rothmund-Thomson syndrome without mosaic trisomy 8, but with structural chromosome instability

##### Stefan Müller^1^, Ilona Kutschka^1^, Yasmin Mehraein^1^, Luana Niculescu^2^, Kathrin Giehl^2^, Ortrud Steinlein^1^

###### ^1^Ludwig-Maximilians-University Munich Institute of Human Genetics, University Hospital Munich, Germany; ^2^Ludwig-Maximilians-University Munich Department of Dermatology, University Hospital Munich, Germany

####### **Correspondence:** Stefan Müller

Rothmund-Thomson syndrome (RTS) is a rare autosomal recessively transmitted genodermatosis associated with congenital bone defects and increased risk of osteosarcoma and skin cancer. RTS is caused by homozygous or compound heterozygous RECQL4 mutations. RecQ helicases are implicated in damaged DNA replication fork repair and are assumed to play roles in the maintenance of genome stability.

Some previous cytogenetic studies described mosaic trisomy 8 in RTS patients, an intriguing finding because RECQL4 is located on 8q24.3. However, it is unknown if these findings point towards a causal association because the functional implications would be difficult to explain. To address this question, we performed a blinded, unbiased interphase FISH screen for copy number changes of all chromosomes and M-FISH karyotyped more than 200 metaphases from a RTS patient.

Our interphase FISH analysis provided no evidence for a general increase in aneuploidy, and specifically no trisomy 8 rates above the cut-off (P < =0.005) were observed. In contrast, M-FISH karyotyping revealed a 2.7% translocation rate in our patient, representing a significant and up to 5.7x increase compared to baseline frequencies in healthy age matched controls from the literature.

We conclude that aneuploidy, and in particular mosaic trisomy 8 is not an apparent pathogenic mechanism in the RTS patient we have analysed. However, and in line with the proposed function of RECQL4 in DNA replication and genome surveillance, we found evidence that increased structural genome instability might be associated with the RTS phenotype.

### 2. Tumour Cytogenetics

#### 2.P42 Clinical and biological significance of Y chromosome loss in a series of 2,423 male patients with Myelodysplastic syndromes and Chronic Myelomonocytic Leukemia

##### Dolors Costa^1^, Meritxell Nomdedeu^2^, Arturo Pereira^3^, Xavier Calvo^4^, Joan Colomer^1^, Francesc Soler^5^, Elisa Luño^6^, Jose Cervera^7^, Amparo Arias^1^, Cándida Gómez^1^, Magui Ortega^8^, Rosa Collado^9^, Isabel Granada^10^, Guillermo Sanz^7^, Jordi Esteve^3^, Elias Campo^1^

###### ^1^Hospital Clínic Unitat D'hematopatologia Barcelona, Spain; ^2^Hospital Plató Hematologia Barcelona, Spain; ^3^Hospital Clínic Hematologia Barcelona, Spain; ^4^Hospital del Mar Hematologia Barcelona, Spain; ^5^Hospital Trias I Pujol Josep Carreras Leukemia Research Institute Badalona, Spain; ^6^Hospital Central de Asturias Hematologia Oviedo, Spain; ^7^Hospital La Fe Hematologia Valencia, Spain; ^8^Hospital Vall D'hebron Hematologia Barcelona, Spain; ^9^Hospital General Hematologia Valencia, Spain; ^10^Hospital Trias I Pujol Hematologia Badalona, Spain

####### **Correspondence:** Dolors Costa

Isolate loss of chromosome Y (-Y) in myelodysplastic syndromes (MDS) is associated to a better outcome but it is also well described as an age-related phenomenon. In this study we aimed to analyze the prognostic impact of -Y in the context of the IPSS-R cytogenetic classification, evaluate the clinical significance of the percentage of metaphases with isolated -Y, and test whether finding -Y may predispose to over-diagnose MDS in patients with borderline morphological features. We evaluated 3,581 male patients from the Spanish MDS Registry with a diagnosis of MDS or chronic myelomonocytic leukemia (CMML). -Y was identified in 177 patients (4.9%). Compared with the 2,246 male patients with normal karyotype, -Y group showed a reduced risk of leukemic transformation that did not translate into a survival advantage. The overall survival and the risk of leukemic transformation were not influenced by the percentage of metaphases with -Y. The -Y group was not enriched in patients with minor morphologic traits of dysplasia, suggesting that the better outcome in the -Y group cannot be explained by enrichment in cases misdiagnosed as MDS. In conclusion, our results support the current recommendation of classifying patients with -Y within the very good risk category of the IPSS-R for MDS and rule out a selection bias as a possible explanation of this better outcome. An analysis of the molecular basis of MDS with isolated -Y would be of interest as it may provide a biological basis of protection against progression to acute leukemia.

